# Impact of Legal Protection on Life-Support Interventions and 3-Month Mortality in the Intensive Care Unit: The Vulne-Rea Study

**DOI:** 10.3390/healthcare14142105

**Published:** 2026-07-14

**Authors:** Jean-Pierre Quenot, Eléa Ksiazek, Isabelle Fournel, Anne-Sophie Mariet, Léa Lerosey, Marine Jacquier, Ludivine Garrier, Nicolas Meunier-Beillard, Fiona Ecarnot, Marie Labruyère, Alicia Taha, Pascal Andreu, Jean-Baptiste Roudaut, Thomas Maldiney, Jean-Philippe Rigaud

**Affiliations:** 1 Service de Médecine Intensive-Réanimation, Centre d’Investigation Clinique 1432, Centre de Recherche Translationnelle en Médecine Moléculaire, Unité Mixte de Recherche 1231, Institut National de la Santé et de la Recherche Médicale, Faculté des Sciences de Santé, Université Bourgogne Europe, Centre Hospitalier Universitaire Dijon Bourgogne, 21000 Dijon, France; lea.lerosey@chu-dijon.fr (L.L.); marine.jacquier@chu-dijon.fr (M.J.); ludivine.garrier@chu-dijon.fr (L.G.); marie.labruyere@chu-dijon.fr (M.L.); alicia.taha@chu-dijon.fr (A.T.); pascal.andreu@chu-dijon.fr (P.A.); jean-baptiste.roudaut@chu-dijon.fr (J.-B.R.); 2 Espace de Réflexion Éthique Bourgogne Franche-Comté (EREBFC), 21000 Dijon, France; 3 Module Épidémiologie Clinique, Centre d’Investigation Clinique 1432, Centre Hospitalier Universitaire Dijon Bourgogne, Institut National de la Santé et de la Recherche Médicale, Université Bourgogne Europe, 21000 Dijon, France; elea.ksiazek@chu-dijon.fr (E.K.); isabelle.fournel@u-bourgogne.fr (I.F.); nicolas.meunier-beillard@u-bourgogne.fr (N.M.-B.); 4 Service de Biostatistiques et d’Information Médicale, Centre Hospitalier Universitaire Dijon Bourgogne, Université Bourgogne Europe, 21000 Dijon, France; anne-sophie.mariet@chu-dijon.fr; 5 Unité de Soutien Méthodologique à la Recherche, Direction de la Recherche Clinique et de l’Innovation, Centre Hospitalier Universitaire Dijon Bourgogne, Université Bourgogne Europe, 21000 Dijon, France; 6 SINERGIES—L’Ingénierie de la Santé, de la Molécule au Patient, Unité de Recherche 4662, Université Marie & Louis Pasteur, Centre Hospitalier Universitaire Besançon Franche-Comté, 25000 Besançon, France; fiona.ecarnot@umlp.fr; 7 Réanimation–Surveillance Continue, Centre Hospitalier William Morey, Université Bourgogne Europe, 71100 Chalon-sur-Saône, France; thomas.maldiney@ch-chalon71.fr; 8 Réanimation–Unité de Surveillance Continue, Centre Hospitalier de Dieppe, 76200 Dieppe, France; jrigaud@ch-dieppe.fr; 9 Espace de Réflexion Éthique de Normandie, Centre Hospitalier Universitaire Caen Normandie, 14000 Caen, France

**Keywords:** intensive care unit, legal protection, vulnerable populations, ethics, mortality

## Abstract

**Background/Objectives**: Adults under legal protection experience management delays and prolonged hospitalization. This study investigated whether ICU outcomes differ between patients with versus without legal protection. **Methods**: This retrospective, single-center study evaluated patients admitted to a French ICU (July 2015–July 2023). Protected patients were compared to autonomous controls matched 1:3 by age and admission year. **Results**: Of the 1051 patients included, 266 (25.3%) were under legal protection. The protected group had a higher proportion of women (43.2% vs. 35.7%, *p* = 0.025), received renal replacement therapy less frequently (8.6% vs. 15.4%, *p* = 0.007), and required non-invasive ventilation more frequently (25.2% vs. 17.2%, *p* = 0.004) than controls. Three-month mortality was 38.7% in controls versus 33.1% in protected patients, showing no significant difference after multivariable adjustment for 6 prespecified clinical variables (aOR 0.816, 95% CI 0.564 to 1.180). In the adjusted model, higher SAPS II scores and vasopressor use were significantly associated with increased 3-month mortality, whereas non-invasive ventilation and non-respiratory admission indications (sepsis, renal failure, trauma) were associated with decreased mortality. **Conclusions**: No significant association was observed between the presence of a legal protection measure prior to ICU admission and 3-month patient mortality. However, these results must be interpreted with caution due to a potential lack of statistical power. Additionally, legally protected patients were observed to receive renal replacement therapy less frequently, whereas non-invasive ventilation was utilized more frequently. Within this context, further research is required to evaluate the impact of legal protection status on the formal collegial deliberations that lead to decisions regarding the limitation of life-sustaining treatments.

## 1. Introduction

In the intensive care (ICU) setting, multiple independent prognostic factors associated with mortality have been well documented in the literature, including specific features of septic shock [1], age and post-ICU trajectories [2,3,4,5], and frailty [6,7]. Beyond these clinical indicators, factors affecting clinical outcomes of patients admitted to the ICU include social inequalities in health and underlying socioeconomic vulnerabilities, which vary widely from country to country [8,9,10].

It has been shown that health inequity can contribute to an increased risk of ICU admission [11], with more severe disease [12,13,14], and a less favourable short-term prognosis both in the ICU [15] and after ICU discharge [16]. Consequently, very elderly patients and/or those presenting with psychiatric or cognitive disorders represent a highly vulnerable population. Within a framework of cumulative vulnerability, lower socioeconomic status heavily drives the long-term progression of such functional and cognitive limitations [17], which in turn intersect with socioeconomic marginalization to dictate poorer health outcomes and adverse clinical trajectories [18]. Indeed, these patients frequently present a higher burden of chronic comorbidities, which may increase their risk of losing long-term autonomy after hospital discharge [19]. Particularly in cases of social isolation or family conflict, legal protection status often formalizes this intersection, as the implementation of legal protection measures may potentially accentuate or exacerbate these social inequalities in health [20].

This risk is compounded by the intersection of social and procedural factors. While legal protection measures fundamentally aim to safeguard vulnerable individuals and promote health equity, their implementation within the ICU introduces distinct procedural challenges. By necessitating a surrogate decision-maker, this legal framework [21] moves the patient’s agency to a third party. In the context of the ICU, this means the guardian is tasked with upholding the patient’s wishes and values at times when the patient is no longer able to express themselves, a role that becomes particularly critical during formal collegial end-of-life deliberations [22].

On a national scale, in France, in 2022, there were approximately 800,000 adults who were under legal protection of some form (guardianship, curatorship, safeguarding of justice or family mediation) [17]. However, the impact of this status on ICU outcomes remains poorly understood. For example, a recent review [23] of 83 articles on factors influencing decision making in the ICU made no mention of patients under legal protection of any kind. Previous studies with small sample sizes and examining very specific situations (end-of-life, dementia) reported that patients under legal protection often have delayed management and decision-making (due to family conflict or limited authority of the guardian), and this in turn can lead to prolonged hospital stays [24,25].

Building on these concerns regarding procedural delays and social disparities, and despite the protective nature of these legal measures, we hypothesized that legal protection could be an independent risk factor for 3-month mortality. Specifically, we posited that these measures alter care management and prolong lengths of stay, thereby driving poorer clinical outcomes and health inequities compared to unprotected patients.

## 2. Methods

### 2.1. Study Design and Population

The Vulne-Rea study is a retrospective, observational, single-centre study comparing the management and outcomes of adults under legal protection with those of adults without legal protective measures in place, among a population of patients admitted to the ICU of the University Hospital of Dijon, France, between 1 July 2015 and 1 July 2023.

Adult patients with legal protective measures in place were identified from individual medical records (as described in Section 2.3). Each patient confirmed to be under legal protection was matched with 3 patients who were not under legal protection and drawn at random from the overall population, identified from the hospital discharge database (Programme de Médicalisation des Systèmes d’Information (PMSI)). Matching was performed using a caliper of ±1 year for age and exact matching for the hospitalization year. Controls were drawn sequentially without replacement. For every patient under legal protection, a first control was drawn randomly among eligible controls. Once each protected patient was matched with a first control, a second random draw was performed among the remaining controls. Finally, a third random draw followed the same procedure to complete the 1:3 ratio.

If any patient had multiple hospital stays during the study period, only one stay was selected randomly for inclusion in the analysis. In case of multiple ICU stays, only the first ICU stay was considered, as it was considered to correspond to the initiation of management. Patients with missing data regarding the primary outcome (3-month mortality) were excluded from the analysis.

In France, judicially mandated legal protection measures are established when an individual’s faculties are impaired, rendering them incapable of defending their own interests. Depending on the patient’s level of autonomy, a judge appoints a third-party representative under four distinct frameworks: safeguarding of justice (temporary, minimal restrictions), curatorship (assistance for major financial decisions), guardianship (continuous and full representation for all administrative acts), or family mediation (direct representation by a close relative). A detailed description of these specific legal structures is available in the Supplementary Materials.

### 2.2. Outcome Measures

The primary endpoint was mortality at 3 months after admission to ICU. Mortality data were retrieved for all patients from the hospital medical records, other referring physicians, or from the national statistics office database of death records (Institut National de la Statistique et des Études Économiques, INSEE; https://www.insee.fr/fr/information/4190491 (accessed on 18 May 2026)).

Secondary endpoints were the length of ICU stay (in days) and the length of hospital stay (in days), which were also retrieved from the PMSI.

The date of this initial ICU admission was defined as the index date for all subsequent outcome evaluations.

### 2.3. Variables of Interest and Data Collection

The existence of legal protection was investigated by two dedicated research assistants in the ICU for all patients during the study period, from the patients’ medical records. In the French public healthcare system, patients (or their representatives) are systematically asked about the existence of legal protection during the admissions process, and the information is noted in the medical file. Other data recorded were obtained from PMSI database and include demographic characteristics, the indication for admission to ICU, the type of legal protection in place, the initial severity of disease as assessed by the Simplified Acute Physiology Score (SAPS) II score [26], and need for life-support therapies in the ICU (mechanical ventilation, non-invasive ventilation, vasopressors, renal replacement therapy).

### 2.4. Sample Size Calculation

Based on a hypothesis of 40% mortality at 3 months after admission to ICU in patients with no legal protection [12,27], 80% power and an alpha risk of 5%, and considering that each patient with legal protection was matched with 3 patients without legal protection, we estimated that 1047 patient would be required in total (of whom 262 under legal protection), to detect a 10% difference in mortality between groups at 3 months after admission to ICU (i.e., a 50% mortality rate in patients under legal protection).

### 2.5. Statistical Analysis

Quantitative variables are described as mean ± standard deviation or median [quartile Q1, Q3] depending on the normality of the distribution, and categorical variables are described as number (percentage). To take the matching into account, patients with versus without legal protection were compared using conditional logistic regression, adjusted for age. Since matching was performed based on the exact year of ICU admission, this variable is inherently controlled by the study design. The impact of legal protection on 3-month mortality after ICU admission was analysed using conditional logistic regression, adjusted for age in model 1. In a second model, we also adjusted for prespecified clinically relevant variables (sex, use of vasopressors, renal replacement therapy, type of ventilation, SAPS II score, and the indication for admission to ICU). This model natively accommodated the unequal matched set sizes. The log-linearity assumption for the continuous variables (age and SAPS II score) was tested using the following procedure: a bivariate conditional logistic regression was performed with and without spline transformation, and the AICs obtained with each model were then considered; if the AIC of the model with spline transformation was at least 5 points lower than the AIC of the model without spline transformation, then the log-linearity assumption was invalid. For both variables, the log-linearity assumption was found to be valid. The spline transformation was performed using a segmentation based on 3 knots placed at the first, second, and third quartiles of the distribution of the variable under consideration. The number of adjustment variables included in the multivariate model respects a ratio of at least 10 events per parameter to be estimated. Interactions between legal protection and age, sex, and SAPS II were tested and not significant at the 0.05 threshold.

As the length of ICU and hospital stay were not normally distributed, and as the Wilcoxon signed-rank test is not adapted due to 3 to 1 matching, the impact of legal protection on these outcomes was examined using the Wilcoxon test. Consequently, these secondary analyses must be considered exploratory. A *p*-value < 0.05 was considered significant for all analyses. Analyses were performed using SAS version 9.4 (SAS Institute Inc., Cary, NC, USA).

## 3. Results

**Figure 1:**
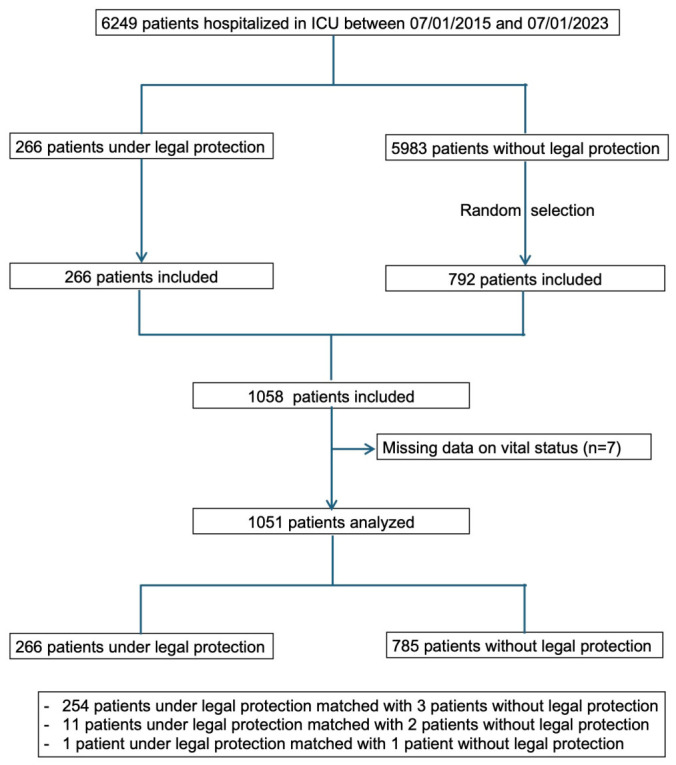
Study flowchart of patient selection, inclusion, and matching process.

During the study period, 6249 patients were admitted to the ICU, of whom 266 were under legal protection and were all included in the present study, and 792 controls were randomly selected. Seven controls without information on the primary endpoint (3-month mortality) were excluded, leading to 785 controls and 1051 patients analyzed. (See Figure 1).

The majority of legally protected individuals were under guardianship or curatorship (respectively 45.9% [*n* =122] and 48.9% [*n* = 130], while safeguarding of justice and family mediation were less common (3.4%, *n* = 9 and 1.9%, *n* = 5 respectively). Patient characteristics were comparable across the different types of legal protection, except for age, which was lower in patients under guardianship and curatorship than in those with other types of protection (Table S1).

The baseline characteristics of the final study population of 1051 patients are described in Table 1. There were significantly more women among those under legal protection (43.2% vs. 35.7%, *p* = 0.025). Patients under legal protection less often required renal replacement therapy (8.6% vs. 15.4%, *p*= 0.007) and more often required non-invasive ventilation (25.2% vs. 17.2%, *p* = 0.004). There was no significant difference in the severity of disease or need for mechanical ventilation between groups.

### Outcomes

The median length of ICU stay was 3 days (IQR [1, 17]) for patients under legal protection compared with 3 days (IQR [2, 6]) for matched controls (*p* = 0.091). Similarly, the median length of hospital stay was 10 days (IQR [4, 23]) for patients under legal protection compared with 9 days (IQR [4, 20]) for matched controls (*p* = 0.068).

Among the 1051 patients, 37.3% died in the 3 months after admission to ICU and 33.1% among those with legal protection (*n =* 266), versus 38.7% among those without legal protection in place (*n =* 785). Accordingly, the existence of legal protection measures was not significantly associated with 3-month mortality, after adjustment (aOR 0.816, 95% CI: 0.564 to 1.180, *p* = 0.280). However, a higher SAPS II score (aOR [95% CI]: 1.047 [1.034; 1.059, *p* < 0.001]) and the use of vasopressors (aOR [95% CI]: 2.115 [1.363; 3.284, *p* = 0.001]) were significantly associated with increased 3-month mortality, whereas the use of non-invasive ventilation was associated with decreased mortality (aOR [95% CI]: 0.596 [0.370; 0.961], *p* = 0.034). The indication for ICU admission (*p* = 0.009) was also independently associated with the main outcome (Table 2).

## 4. Discussion

To our knowledge, this is the first matched cohort study to examine 3-month ICU mortality across all forms of legal protection in the French system. This work follows the logical progression of research conducted by our group regarding the influence of socioeconomic vulnerability on patient care trajectories and prognosis both during and after intensive care [12,28]. Our study demonstrates that patients under legal protection do not have a statistically different 3-month mortality compared to those without such measures, even after multivariate analysis accounting for standard mortality risk factors.

This lack of statistical difference in prognosis may be observed because, despite well-documented healthcare inequalities in France [29], we hypothesize that the national social security system acts as a structural equalizer. By providing universal core coverage and 100% reimbursement for severe or chronic illnesses, this system could largely mitigate financial barriers to high-intensity diagnostic and therapeutic interventions. Consequently, ICU care quality might remain largely decoupled from a patient’s socioeconomic status across the French territory. However, our study did not directly measure any variable related to insurance coverage, reimbursement, or financial access, meaning that this structural mechanism remains a hypothesis that cannot be formally verified with our dataset. Future international studies are warranted to compare our findings with healthcare systems in countries such as the United States or the United Kingdom, where the legal implications of guardianship and surrogacy differ significantly from the French framework.

This is supported by the IVOIRE study [12], where 48.6% of the 1 294 patients were classified as “deprived” according to the EPICES score—the French reference for evaluating socioeconomic deprivation—and showed a 3-month mortality rate of 31.6% compared to 30.5% in non-deprived patients, with no statistical difference between the two groups. Similarly, the RECOVIDS study [28], which evaluated long-term outcomes among survivors of COVID-19 ICU admission (where deprived patients accounted for 40.0% of the population), found no significant disparities in respiratory sequelae at 6 months, affecting 81.0% of deprived patients versus 78.0% of non-deprived patients.

Other authors [13,30] suggest a very strong link between the socioeconomic precarity of patients admitted to the ICU and their prognosis. Proposed explanations for this deprivation-led disparity include geographic distance or more difficult access to the healthcare system, as well as a tendency to wait longer before seeking treatment, particularly for patients living alone at home [31]. A recent review of the literature [15], it showed that a low socioeconomic level is associated with higher mortality after intensive care; the primary explanation provided is the disparity between healthcare systems worldwide, even among developed countries [32,33]. However, these studies focus strictly on socioeconomic deprivation, whereas our work specifically evaluates legal protection status, which serves as a formal marker of an individual’s overall vulnerability rather than a direct constituent of financial precarity itself.

The French legal framework governing the protection of vulnerable adults [21,22] provides a strict mandate for a designated protector to ensure the security, health, and fundamental needs of the individual. By requiring that healthcare choices respect the patient’s values and preferences through this surrogate mechanism, the legislation effectively standardizes the management of protected and non-protected patients alike. This legal oversight likely contributes to a more homogeneous care trajectory across the ICU population, regardless of individual socioeconomic status.

Another particularly interesting result of our study is that patients under legal protection measures received renal replacement therapy less frequently and were more often managed with non-invasive ventilation. These results should be interpreted with caution as they could be explained by different hypotheses that our study cannot formally disentangle. On one hand, this pattern may reflect a substantial indication bias due to the retrospective nature of our data collection, which does not allow for full adjustment for the clinical context. On the other hand, these findings could alternatively reflect a deliberate tailoring of life-support therapies. In these instances, the formal collegial deliberations among the ICU team and with the legal surrogate might have weighed the patient’s extreme frailty and comorbidities against their established care goals. Although our study lacks these specific elements, the parity in 3-month mortality suggests that these decisions—made in accordance with professional guidelines and the legal framework [22,34,35,36,37]—might reflect an attempt at appropriate, personalized management rather than a deficit in care.

Moreover, non-invasive ventilation (NIV) was independently associated with a lower risk of 3-month mortality. This protective association likely reflects an indication bias, as NIV is typically reserved for less severe respiratory failure, thereby avoiding endotracheal intubation and its associated risks, such as sedative-related complications and ventilator-associated pneumonia. This is consistent with previous literature showing highly variable effects of NIV depending on the underlying patient severity and clinical presentation [38].

Regarding patient preferences, specific data regarding patient wishes—expressed either directly or through written or oral advance directives—were unavailable for this cohort. This underscores a critical area for future research, particularly regarding how ICU professionals perceive and integrate legal protection status during formal collegial deliberations focused on the appropriate level of life-support therapies.

The existence of a legal protection measure does not preclude the presence of relatives who can support the patient, act as interlocutors with the medical team, and communicate the patient’s wishes. In this context, the protection measure serves as a formal marker of the protected person’s vulnerability rather than a constituent of that vulnerability itself; as such, it does not strictly encompass the domain of medical decision-making or dictate clinical management. Since protected patients did not exhibit higher mortality, it is reasonable to suggest that a patient’s legal status does not bias the trajectory of ICU care. Conversely, whether these protective measures exert a more significant impact on longer-term outcomes remains a hypothesis to be tested, as our study did not collect functional or trajectory data beyond 3 months. Evaluating these long-term supportive effects represents a direction for future research.

### Strengths and Limitations

This study has some limitations. First, it is a single-centre, retrospective study. The results likely cannot be generalized to other French hospitals or to other countries with differing healthcare systems.

Second, due to its retrospective design, the analyzed data were strictly limited to the information routinely collected in medical records or the medical information system. Consequently, we could not account for specific confounding factors or include variables such as the Charlson Comorbidity Index, the Clinical Frailty Scale, or precise cognitive disability status, as these are not systematically reported in routine medical files. Furthermore, because matching was strictly restricted to age and hospitalization year, and despite adjusting for severity at admission and life therapy treatment in our multivariate models, residual confounding remains a limitation.

Third, our study only accounts for patients with a formalized legal status; we were unable to identify vulnerable patients for whom a protection measure might have been indicated but not yet legally implemented.

Fourth, our study might be underpowered to detect the mortality difference that was actually observed, introducing a risk of a type II statistical error. Our initial sample size calculation assumed a 40% mortality rate in the unprotected control group and a 50% mortality rate in the legally protected group. However, while the observed control mortality of 38.7% was near our initial 40% hypothesis, the observed mortality in the protected group was 33.1%, which was substantially lower than the expected 50%. Consequently, the study was powered for a much larger difference than the 5.6% difference observed. Therefore, our non-significant primary result must be interpreted with caution as a possible failure to detect a true difference rather than definitive evidence that no effect exists.

Finally, due to the retrospective nature of our database, we lacked granular data regarding the precise involvement and documented interactions of guardians or legal representatives during clinical deliberations. We cannot rule out a potential interaction between the existence of legal protection, and the other factors, such as decisions not to initiate life-support therapy in patients under legal protection, thus influencing 3-month mortality.

Our study also has some strengths. First, the primary outcome information had a high level of exhaustiveness through the cross-checking of several data sources such as the PMSI (medical information system), medical files, and the national death registry. Second, although the final observed mortality rates deviated from our initial hypotheses, our sample size estimation was transparently rooted in a comprehensive review of the available literature. Third, the matching process allowed us to control for potential confounders regarding 3-month mortality. Nevertheless, we cannot exclude the possibility that a longer follow-up (e.g., 6 or 12 months) would reveal differences between groups, as the long-term impact of ICU admission is often observed long after the fact [12,28]. Finally, only 7 patients (<1%) were excluded from the final analysis due to missing data on the primary endpoint, which minimizes any attrition bias and reinforces the validity of our observed results.

## 5. Conclusions

In conclusion, our study found no significant association between the presence of a legal protection measure prior to ICU admission and 3-month patient mortality (33.1% in the legally protected group versus 38.7% in the unprotected group; aOR 0.816, 95% CI 0.564–1.180). However, these results must be interpreted with caution due to a potential lack of statistical power. Additionally, legally protected patients were observed to receive renal replacement therapy less frequently, whereas non-invasive ventilation was utilized more frequently. Within this context, further research is required to evaluate the impact of legal protection status on the formal collegial deliberations that lead to decisions regarding the limitation of life-sustaining treatments.

## Figures and Tables

**Table 1:** Baseline characteristics at admission to the ICU in patients with vs. without legal protection measures in place.

	All (*n* = 1051)	No Protection (*n* = 785)	Legal Protection (*n* = 266)	*p*
Female gender	395 (37.6)	280 (35.7)	115 (43.2)	0.025
SAPS II	47.9 ± 21.3	48.3 ± 22.1	46.7 ± 18.8	0.321
Indication for admission in ICU				0.047
Sepsis	57 (5.4)	40 (5.1)	17 (6.4)	
Neurological	87 (8.3)	55 (7)	32 (12)	
Cardiac and shock	180 (17.1)	148 (18.9)	32 (12)	
Respiratory	398 (37.9)	294 (37.5)	104 (39.1)	
Gastro-enterology	89 (8.5)	73 (9.3)	16 (6)	
Renal	77 (7.3)	56 (7.1)	21 (7.9)	
Trauma	79 (7.5)	60 (7.6)	19 (7.1)	
Other reasons ^a^	84 (8)	59 (7.5)	25 (9.4)	
Vasopressors	548 (52.1)	409 (52)	139 (52.3)	0.808
Invasive mechanical ventilation	634 (60.3)	483 (61.5)	151 (56.8)	0.185
Non-invasive ventilation	202 (19.2)	135 (17.2)	67 (25.2)	0.004
Renal replacement therapy	144 (13.7)	121 (15.4)	23 (8.6)	0.007

*p*-values were obtained from conditional logistic regressions, adjusted for age and year of hospitalization; outcome was legal protection status. Boldface indicates *p*-value < 0.05. ^a^ Other indications: Tumor (*n* = 16, 15 cancers); Blood disorders (*n* = 5); Endocrine, nutritional or metabolic disorders (*n =* 21); Psychiatric or behavioural disorders (*n =* 16); Skin and subcutaneous tissue diseases (*n =* 4); Disorders of joints, muscles or connective tissue (*n =* 13); Pregnancy, childbirth, postpartum (*n =* 1); Congenital malformations and chromosomal anomalies (*n =* 1); symptoms, signs and abnormal laboratory results not otherwise classified: Coma and other non-classified shock (*n =* 5); Other surgical follow-up (*n =* 2).

**Table 2:** Multivariable conditional logistic regression analysis of factors associated with 3-month mortality after ICU admission.

	Model 1		Model 2	
	OR [95% CI]	*p*-Value	OR [95% CI]	*p*-Value
Legal protection	0.763 [0.561; 1.037]	0.084	0.816 [0.564; 1.180]	0.280
Female sex			0.799 [0.545; 1.172]	0.251
Vasopressors			2.115 [1.363; 3.284]	0.001
Renal replacement therapy			1.414 [0.824; 2.427]	0.208
Invasive mechanical ventilation			0.728 [0.458; 1.156]	0.179
Non-invasive mechanical ventilation			0.596 [0.370; 0.961]	0.034
SAPS II			1.047 [1.034; 1.059]	<0.001
Indication for admission to ICU				0.009
Sepsis			0.365 [0.169; 0.786]	
Neurological			0.507 [0.242; 1.064]	
Cardiac and shock			0.846 [0.503; 1.424]	
Gastro-enterology			0.695 [0.352; 1.371]	
Renal			0.315 [0.150; 0.663]	
Trauma			0.333 [0.132; 0.837]	
Other reasons			0.648 [0.313; 1.339]	
Respiratory			Reference	

Model 1: conditional logistic regression adjusted for age. Model 2: Model 1 further adjusted for sex, use of vasopressors, renal replacement therapy, type of ventilation, SAPS II score, and the indication for admission to ICU. OR, odds ratio; 95%CI, 95% confidence interval; SAPS II, Simplified Acute Physiology Score; ICU, intensive care unit. For both models: N = 1051 patients, 392 died. Boldface indicates *p*-value < 0.05.

## Data Availability

All the data arising from this study are reported in the results and tables of this manuscript. Further inquiries can be directed to the corresponding author.

## Supplementary Materials

The following supporting information can be downloaded at https://www.mdpi.com/article/10.3390/healthcare14142105/s1, Table S1: Patient characteristics by type of legal protection.
