# Supplementary material for: Impact of Legal Protection on Life-Support Interventions and 3-Month Mortality in the Intensive Care Unit: The Vulne-Rea Study
Source: Healthcare (Basel). 2026 Jul 14;14(14):2105. doi: 10.3390/healthcare14142105 (PMC13411860; doi:10.3390/healthcare14142105)
Supplement: Supplementary file 1 [file healthcare-14-02105-s001.zip › healthcare-4359005-supplementary.pdf]

Table S1. Patient characteristics by type of legal protection.

Variable Value	All N=266	Guardianship N=122	Curatorship N=130	others N=14	P-value
Female sex	115 (43.2)	53 (43.4)	57 (43.8)	5 (35.7)	0,8393
Age (years) (median [Q1 ; Q3])	60.5 [53 ; 71]	60 [50 ; 70]	60 [53 ; 70]	71.5 [67 ; 80]	**0,007**
SAPS II (median [Q1 ; Q3])	44.5 [33 ; 56]	45 [33 ; 58]	43.5 [33 ; 55]	49 [35 ; 68]	0,559
Vasopressors	139 (52.3)	61 (50.0)	71 (54.6)	7 (50.0)	0,753
Renal replacement therapy	23 (8.6)	9 (7.4)	12 (9.2)	2 (14.3)	0,672
Invasive mechanical ventilation	151 (56.8)	76 (62.3)	70 (53.8)	5 (35.7)	0,106
VNI	67 (25.2)	31 (25.4)	35 (26.9)	1 (7.1)	0,192
Indication for admission to ICU					-
Sepsis	17 (6.4)	5 (4.1)	10 (7.7)	2 (14.3)	
Neurological	32 (12.0)	20 (16.4)	11 (8.5)	1 (7.1)	
Cardiac and shock	32 (12.0)	14 (11.5)	16 (12.3)	2 (14.3)	
Respiratory	104 (39.1)	52 (42.6)	47 (36.2)	5 (35.7)	
Gastro-enterology	16 (6.0)	6 (4.9)	9 (6.9)	1 (7.1)	
Renal	21 (7.9)	8 (6.6)	11 (8.5)	2 (14.3)	
Trauma	19 (7.1)	7 (5.7)	12 (9.2)	0 (0)	
Other reasons	25 (9.4)	10 (8.2)	14 (10.8)	1 (7.1)	
Inclusion period					-
2015	20 (7.5)	14 (11.5)	6 (4.6)	0 (0.0)	
2016	31 (11.7)	17 (13.9)	13 (10.0)	1 (7.1)	
2017	31 (11.7)	15 (12.3)	16 (12.3)	0 (0.0)	
2018	41 (15.4)	18 (14.8)	20 (15.4)	3 (21.4)	
2019	36 (13.5)	9 (7.4)	25 (19.2)	2 (14.3)	
2020	28 (10.5)	11 (9.0)	16 (12.3)	1 (7.1)	

2021	26 (9.8)	14 (11.5)	10 (7.7)	2 (14.3)	
2022	34 (12.8)	18 (14.8)	14 (10.8)	2 (14.3)	
2023	19 (7.1)	6 (4.9)	10 (7.7)	3 (21.4)	

Others: safeguarding of justice (n=9), family mediation (n=5)

Qualitative variables: Chi-square test. Quantitative variables:

Wilcoxon test, -: test not applicable, boldface indicates p-value <0,05
